# GAN-based data augmentation for transcriptomics: survey and comparative assessment

**DOI:** 10.1093/bioinformatics/btad239

**Published:** 2023-06-30

**Authors:** Alice Lacan, Michèle Sebag, Blaise Hanczar

**Affiliations:** IBISC, University Paris-Saclay (Univ. Evry), Evry 91000, France; TAU, CNRS-INRIA-LISN, University Paris-Saclay, Gif-sur-Yvette 91190, France; IBISC, University Paris-Saclay (Univ. Evry), Evry 91000, France

## Abstract

**Motivation:**

Transcriptomics data are becoming more accessible due to high-throughput and less costly sequencing methods. However, data scarcity prevents exploiting deep learning models’ full predictive power for phenotypes prediction. Artificially enhancing the training sets, namely data augmentation, is suggested as a regularization strategy. Data augmentation corresponds to label-invariant transformations of the training set (e.g. geometric transformations on images and syntax parsing on text data). Such transformations are, unfortunately, unknown in the transcriptomic field. Therefore, deep generative models such as generative adversarial networks (GANs) have been proposed to generate additional samples. In this article, we analyze GAN-based data augmentation strategies with respect to performance indicators and the classification of cancer phenotypes.

**Results:**

This work highlights a significant boost in binary and multiclass classification performances due to augmentation strategies. Without augmentation, training a classifier on only 50 RNA-seq samples yields an accuracy of, respectively, 94% and 70% for binary and tissue classification. In comparison, we achieved 98% and 94% of accuracy when adding 1000 augmented samples. Richer architectures and more expensive training of the GAN return better augmentation performances and generated data quality overall. Further analysis of the generated data shows that several performance indicators are needed to assess its quality correctly.

**Availability and implementation:**

All data used for this research are publicly available and comes from The Cancer Genome Atlas. Reproducible code is available on the GitLab repository: https://forge.ibisc.univ-evry.fr/alacan/GANs-for-transcriptomics

## 1 Introduction

The growing amount of data and knowledge about genomics is expected to play a crucial role in precision medicine, in particular in precision oncology, where the goal is to develop treatments that target the characteristics of patients’ tumors. Specifically, the information in the transcriptome can lead to better classification of a patient’s cancer and better prediction of their response to treatment. The potential use of this data relies on the measurement of gene expression by RNA sequencing (RNA-Seq) (Makin 2020) and NGS. They allow high-throughput sequencing with both a low cost and high accuracy (Hong et al. 2020). Dedicated methods have also been developed to exploit the data better (Huang et al. 2016). Among the machine learning approaches applied to cancer diagnosis and prognosis prediction tasks (Kourou et al. 2015), deep learning methods are increasingly popular in transcriptomics due to their ability to handle high-dimensional data and learn complex relationships (Libbrecht and Noble 2015; Katzman et al. 2018; Kim et al. 2020).

Deep learning faces a primary limitation in the transcriptomic domain due to data scarcity (Koumakis 2020): it is a *small n large p* problem. More formally, the number *n* of samples remains in the hundreds per pathology (due to the remaining costs of RNA sequencing and the lack of consensus on data integration methodologies) while the number of genes is circa 20 000. The small *n* issue (also hindering diversity and representativity of the data) entails a risk of overfitting for deep learning methods (Halevy et al. 2009) that are notoriously data hungry. For this reason, neural approaches in transcriptomics mostly rely on autoencoders (AE) and multilayer perceptrons (MLPs) (Guo et al. 2017; Danaee et al. 2017; Hanczar et al. 2018; Katzman et al. 2018; Kim et al. 2020).

A research direction, early suggested to handle sparse datasets (Lecun et al. 1998), is based on data augmentation, i.e. the generation and exploitation of artificial data samples expectedly following the same distribution as the true data (Shorten and Khoshgoftaar 2019; Feng et al. 2021). Data augmentation, often based on domain knowledge about label-invariant transformations (Section 2), contributes to the better robustness of the learned models.

When domain knowledge does not provide such invariance operators, most data augmentation approaches rely on model-based methods using deep generative models, such as Variational Auto-Encoders Welling and Kingma (2014) or Generative Adversarial Networks (GANs) Goodfellow et al. (2014) (Section 3).

As commonly acknowledged, there are no such label-invariant operators in transcriptomics, suggesting that deep generative models constitute the only way to achieve data augmentation. Data augmentation is assessed either from a supervised or unsupervised learning perspective. In the former case, improvements in the accuracy or generalization of the model can be measured with respect to augmentation in the training set. In the latter case, generated data quality can be evaluated with specific performance indicators. We propose a range of such indicators in Section 4.1.

This article presents a survey, and a comparative assessment of prominent GAN approaches to data augmentation in transcriptomics. The first contribution of the article is to rigorously compare the reviewed algorithms on the large-sized TCGA dataset (Weinstein et al. 2013) for cancer phenotypes predictions. The discrepancy between the true and the generated data is analyzed, and some interpretation is proposed. Our second contribution is an attention-based GAN model (AttGAN) tailored to data augmentation in transcriptomics, where the attention neural architecture is derived from domain knowledge related to genes and protein relationships.

Deep generative augmentation methods are presented in Section 3, the training and the comparison methodology are detailed in Sections 4 and 5, report and discuss the main results.

## 2 Knowledge-based data augmentation

This section briefly presents the rationale for data augmentation and some early approaches.

An early approach to data augmentation proceeds by adding copies of the original samples perturbed with Gaussian noise. These data augmentation contribute to the robustness of the model w.r.t. the data noise and can be analyzed in terms of regularization with a Gaussian kernel (Grandvalet et al. 1997). Data augmentation is now widely used as a regularization technique in computer vision and natural language processing (Devries and Taylor 2017; Yun et al. 2019; Hendrycks et al. 2020; Uddin et al. 2020).

Knowledge-based data augmentation relies on the known transformation operators, exploiting the label invariance w.r.t. geometric transformations in computer vision (Lecun et al. 1998; Shorten and Khoshgoftaar 2019), or syntax parsing and terms commutation in natural language processing (Feng et al. 2021) or other operators (Devries and Taylor 2017; Yun et al. 2019; Hendrycks et al. 2020; Uddin et al. 2020).

The difficulty of identifying efficient transformation operators limits the application of the approach to structured spaces, e.g. involving time series (Wen et al. 2021) or graph-based data (Zhao et al. 2020). The cost of manually identifying the appropriate transformation operators prompted the emergence of a new research field (Cubuk et al. 2019; Mounsaveng et al. 2020; Ni et al. 2021) aimed at the automatic identification of such operators.

A few authors have proposed a theoretical analysis of data augmentation (Dao et al. 2018; Shao et al. 2022). A key issue is determining whether the augmented data fall within or outside the support of the true data distribution (Shao et al. 2022). Lopes et al. (2021) advocate a careful tradeoff between the likelihood versus diversity of the augmented data w.r.t. the true data distribution.

## 3 Generative model-based data augmentation

*Notations.* Let $D_{t}$ denote the (unknown) true data distribution; the training set is iid sampled according to $D_{t}$. In the following, $D_{g}$ denotes the generated distribution and $N(0,I_{d})$ the d is dimensional (Gaussian) noise distribution.

Deep generative models proceed by learning a distribution $D_{g}$ as close as possible to the target distribution $D_{t}$. These models, currently among the hottest topics in Deep Learning and Machine Learning, are structured along the main two directions pioneered by Variational Auto-Encoders (Welling and Kingma 2014) and GANs (Goodfellow et al. 2014). The formal background of GANs will be presented in the next section before discussing the GAN-based approaches to data augmentation in transcriptomics.

### 3.1 Generative adversarial networks

A GAN involves two components: a generator *G* and a discriminator *D* trained simultaneously. Both components compete with each other in a zero-sum game (Goodfellow et al. 2014). Generator *G* returns $\hat{x}=G(z)$ after the current $D_{g}$ distribution and with *z* sampled after $N(0,I_{d})$. Discriminator *D* is responsible for distinguishing between the true data (*x* in Class 1) and generated data ($\hat{x}$ in Class 0). The joint training of both modules aims at generating a distribution $D_{g}$ such that the discriminator *D* cannot distinguish among $D_{t}$ and $D_{g}$. A GAN, described from the parameters of *G* and *D*, is learned by solving the following min–max optimization problem: where *D*(*x*) is the probability estimated by the discriminator that *x* belongs to the true data.


(1) $$\underset{G}{\min}\underset{D}{\max}L(D,G)=\underset{x∼D_{t}}{E}[\log(D(x)]+\underset{z∼P_{z}}{E}[\log(1-D(G(z))],$$

More convolved generative distributions are obtained by exploiting the data structure, for instance using convolutional architectures (Radford et al. 2016) or domain-transfer terms (Zhu et al. 2017). Conditional GANs (Mirza and Osindero 2014), Wasserstein-GANs (Arjovsky et al. 2017), and attention-based GANs (Zhang et al. 2019) are briefly introduced for the sake of self-containedness.

#### 3.1.1 Conditional GANs

To refine the generation process, the generative model $D_{g}$ can be made conditional to some conditional variables *c* (e.g. age, gender, type of tissue, presence of cancer), defining $D_{g}(|c)$:


$$\underset{G}{\min}\underset{D}{\max}L(D,G)=\underset{x∼D_{t}}{E}[\log(D(x|c)]+\underset{z∼P_{z}}{E}[\log(1-D(G(z|c))]$$

#### 3.1.2 WGAN-GP

GANs face several difficulties due to the nature of the loss and the dynamics of min–max optimization. The loss function [Equation (1)] computes the Jensen–Shannon divergence between $D_{t}$ and $D_{g}$, to be minimized. Unfortunately, in the case where the supports of both distributions do not overlap, this loss yields the maximum Jensen–Shannon divergence, with a null gradient (Salimans et al. 2016; Arjovsky and Bottou 2017); therefore the optimization is stuck in a bad local optimum.

Arjovsky et al. (2017) propose to palliate this drawback by replacing the discriminator component with an estimator of the Wasserstein distance between $D_{g}$ and $D_{t}$, to be minimized. The term $E_{x∼D_{t}}[D(x|c)]+E_{z∼P_{z}}[1-D(G(z|c))]$ in Equation (1) estimates the Wasserstein distance between $D_{g}$ and $D_{t}$; this estimate is exact subject to the gradient of *D* to have unity norm. Eventually, the loss considered in the WGAN-GP reads: with *λ* the penalty weight enforcing *D* to be a 1-Lipschitz function and $\tilde{x}$ linearly interpolated data between true *x* and generated $\hat{x}$.


(2) $$\begin{array}{cc} \underset{G}{\min}\underset{D}{\max} & L(D,G)=E_{x∼D_{t}}[D(x|c)]-E_{z∼P_{z}}[D(G(z|c))]\\ & +\lambda E_{\tilde{x}∼D_{g}}[(||\nabla_{\tilde{x}}D(\tilde{x})|{|}_{2}-1{)}^{2}], \end{array}$$

#### 3.1.3 Attention-based GANs

GANs also borrow the expressive power of attention-based layers to enhance the generator architecture (Zhang et al. 2019). Informally, the self-attention layer is used to polish the primary output of the generator to account for the dependencies among the features (here, the genes). More formally, let $x_{i}^{L-1}$ denote the value of the *i*th gene before the self-attention layer; its value after the self-attention layer is computed as $x_{i}=x_{i}+\gamma \mathrm{att}(x_{i}^{L-1})$, with: where *W*_K_, *W*_Q_, and *W*_V_ (respectively, referred to as key, query, and value) and *γ* are the self-attention parameters to learn. The attention coefficient *α_ij_* models the impact of gene *j* on gene *i*, depending on the context defined by *W*_K_ and *W*_Q_.


$$\{\begin{array}{c} \mathrm{att}(x_{i}^{L-1})=\sum_{j}\alpha_{j,i}W_{V}x_{i}^{L-1}\\ \alpha_{j,i}=\mathrm{softmin}(e_{ij})=\frac{\;\text{exp}(-e_{ij})}{\sum_{k}\;\text{exp}\;(-e_{ik})}\\ e_{ij}=|W_{K}x_{i}^{L-1}-W_{Q}x_{j}^{L-1}| \end{array},$$

In the mainstream attention framework, aimed at computer vision or natural language processing, similarity scores ($e_{i,j}$) are computed as a dot product between d dimensional vectors of encoded pixels (through different channels) or encoded words.

*Attention in transcriptomics*. To our best knowledge, no attention framework has yet been deployed on genomics. In the presented experiments (Section 5), the genes *x_i_* are scalars so the L1-norm is used instead of a dot product as a similarity score. Consequently, softmax-based normalization is replaced by a softmin one.

A key limitation of the attention framework lies in the amount of memory and computational resources, quadratic in the dimension of the input space (20 000 in the transcriptomics case; thus matrices *W*_K_, *W*_Q_, and *W*_V_ hardly fit in memory).

We propose a dedicated attention module (Supplementary Appendix SA), restricting the considered pairs (*i*, *j*) to genes with high correlations (w.r.t. co-expression knowledge) and/or high protein-to-protein interactions (PPI).

### 3.2 Data generation for transcriptomics

As mentioned, most existing genomic datasets, e.g. TCGA, are small by the standards of deep learning, with significant class imbalance and potential biases w.r.t. the test distribution due to pathology rareness, sequencing accessibility, and privacy concerns.

In genomics, generative models are called upon to achieve data augmentation. The Population Scale Genomic approach is based on a conditional-GAN (PG-cGAN) (Chen et al. 2020). Building upon PG-cGAN, the Offspring GAN approach aims to limit mode collapsing and mitigates the impact of training biases in Das and Shi (2022). GANs and restricted Boltzmann machines are investigated by Yelmen et al. (2021) to generate genomes.

In transcriptomics, few studies have been conducted on unsupervised learning and generative models. Early works on single-cell RNA-seq data focus on latent representation and dimensionality reduction. Lopez et al. (2018) introduce single-cell variational inference demonstrating improvement in batch correction, visualization, clustering, and differential expression analysis. Way and Greene (2018) propose to leverage the latent representation of transcriptomic data in variational AEs (VAEs) latent spaces to capture biologically relevant features for cancer-related tasks. Similarly, Wang and Gu (2018) use a VAE for single-cell data reduction. Further work for single-cell applications has been suggested using VAEs (Eraslan et al. 2019; Grønbech et al. 2020) and GANs (Ghahramani et al. 2018; Marouf et al. 2020; Liu et al. 2021). Regarding bulk RNA-seq data generation, Park et al. (2020) propose a GAN to predict Alzheimer’s disease progression. They create augmented samples using linear interpolations but conclude that a systematic data augmentation strategy is required to benefit from gene expression informative power with deep learning. Viñas et al. (2022) deploy a promising WGAN-GP-based (Section 3.1.2) data generation strategy for cancer classification. They obtain state-of-the-art results in cancer classification accuracy when using their generated samples as training samples. Though they propose some analysis of the generated data structure and similarity with the true expression data, they do not provide information on data diversity.

## 4 Experimental setting

As stated, the goal of the article is to investigate and interpret the differences in GAN-based data augmentation methods, depending on architectures, learning criteria, and hyper-parameter settings.

This section first reviews the performance indicators of interest to measure the added value of data augmentation, before discussing the goals of the presented experiments and the experimental setting.

### 4.1 Performance indicators

As said, data augmentation can be assessed in a supervised or unsupervised manner.

#### 4.1.1 Supervised indicators

*Data augmentation gain.* In a supervised learning perspective, the benefit of data augmentation is first measured from the improvement of prediction accuracy on a test set: ΔAcc(n,m)=Acc(n,m)-Acc(n,0) where Acc(*n*, *m*) denotes the predictive accuracy of the model trained from *n* true samples augmented with *m* generated samples.

Another expected benefit of data augmentation is to augment the coverage of the learned model, aimed to a better out-of-distribution generalization. Generated data sufficiently similar to the true training samples distribution should not impair the training error, while sufficiently diversified data should help reduce the generalization error on the true test set. Formally, this augmented coverage is measured by comparing a model performance when it is trained with true data only as opposed to being trained with a combination of true and generated samples.

*Reverse validation* corresponds to evaluating the predictive capacity of the generated data per se. Formally, an MLP trained from generated data is tested on true data (not seen when training the generative model). Intuitively, if the MLPs trained from either true data only, or generated data only, yield same performances on the test true data, this suggests that the generated data do preserve the cancer-related discriminant information.

#### 4.1.2 Unsupervised indicators

The challenge is to assess the quality of the generated data in high dimension.

*Frechet inception distance (FD)* relies on the dimensionality reduction, achieved by mapping sample *x* onto its activations *y* in some latent space, e.g. the latent space of a discriminant neural network (Heusel et al. 2017), under the assumption that true and generated data should have similar activation functions distributions. In computer vision, the FD is computed using the last pooling layer prior to the output classification of images in the Inception v3 pretrained network. Informally, the true and generated distributions are approximated as Gaussian distributions, respectively, noted $N(\mu_{t},\Sigma_{t})$ and $N(\mu_{g},\Sigma_{g})$. FD is the Wasserstein-2 distance between both Gaussian distributions (the lower, the better):


(3) $$\mathrm{FD}(D_{t},D_{g})=||\mu_{t}-\mu_{g}{\left|\right|}^{2}+\mathrm{Tr}\left(\Sigma_{t}+\Sigma_{g}-2\sqrt{\Sigma_{t}.\Sigma_{g}}\right).$$

In computer vision, low FDs appear to be well correlated with higher quality generated images (Heusel et al. 2017). However, Kynkäänniemi et al. (2019) demonstrate that a low FD can still be associated with low-quality images.

As noted by Heusel et al. (2017), FD is sensitive to the number of considered samples. We thus adapt the FD measure to transcriptomics by (i) considering all true training data samples; (ii) adjusting the embedding in the low-dimensional space considered to compute FD.

Two embeddings are used in the experiments (more in Section 4.4): FD-binary is computed based on the last hidden layer of an MLP trained to discriminate cancerous from noncancerous samples; FD tissues are likewise computed based on an MLP discriminating among the diverse 24 tissues.

*Precision and recall* actually measure the distance between $D_{t}$ and $D_{g}$, accounting for the intrinsic dimensionality of their support (c). Formally, precision (respectively, recall) is the probability of a sample from $D_{g}$ (respectively, $D_{t}$) to fall within the support of $D_{t}$ (respectively $D_{g}$) ranging in [0,1]. A high precision suggests that generated data are “close” to true data; a high recall suggests that no true data is “far” from generated data.

Following Kynkäänniemi et al. (2019), a manifold approximation is used to account for the intrinsic dimensionality of the supports. Formally, to each (true or generated) sample *x* is associated a ball $B(x,r_{k}(x))$ with $r_{k}(x)$ the distance between *x* and its *k*th nearest neighbor in $R^{20,000}$. The coverage of these balls approximates the manifold *H* containing *x*. The precision is thus defined as the percentage of generated samples falling in *H_t_* and conversely the recall is the percentage of true samples falling in *H_g_*:


(4) $$\begin{array}{c} \mathrm{precision}(x\in D_{g})=\{\begin{array}{c} 1,\text{iff}\;x\in H_{t}\\ 0,\text{otherwise} \end{array}\\ \mathrm{recall}(x\in D_{t})=\{\begin{array}{c} 1,\text{iff}\;x\in H_{g}\\ 0,\text{otherwise} \end{array} \end{array}.$$

As the curse of dimensionality impacts the relevance of Euclidean distances, we set the number of neighbors *k* to 50 to enforce stable precision and recall measures (Supplementary Appendix SC).

Like FD, the precision and recall are sensitive to the number of samples, and we consider all true samples in estimating these indicators.

Another performance indicator is the F1-score that is the harmonic mean of the precision and recall measures. Analogously to the FD score, these metrics are computed on the true training set to benefit from a large amount of comparison samples as suggested by Kynkäänniemi et al. (2019).

*Adversarial accuracy (AA).* In the context of sensitive data, Yale et al. (2020) developed the AA metric to assess whether the generated data are neither too far from true data (hindering their utility) nor too close (possibly entailing a breach of privacy). Formally, letting $d_{\mathrm{tt}}(i)$ and $d_{\mathrm{tg}}(i)$, respectively, denote the min distance of the *i*th true sample to another true (respectively, generated) sample, and symmetrically, $d_{\mathrm{gt}}(j)$ and $d_{\mathrm{gg}}(j)$ denote the min distance of the *j*th generated sample to another true (respectively, generated) sample, it comes:


(5) $$AA=\frac{1}{2}\left(\frac{1}{n}\sum_{i=1}^{n}1(d_{tg}(i)>d_{tt}(i))+\frac{1}{n}\sum_{i=1}^{n}(d_{gt}(i)>d_{gg}(i))\right).$$

AA can be understood as the accuracy of a 1-NN classifier discriminating true from generated data. When AA decreases to 0, this suggests that the generated data are too close copies of the true data (the generator overfits the training set). On the contrary, an AA close to 1 indicates that the generated data can easily be discriminated from the true data (the generator underfits the training set). Overall, a generative model with an AA around 0.5 should achieve a good tradeoff between accuracy and privacy.

Similar to precision and recall, AA is based on Euclidean distances in the data space; however, it is not sensitive to the number of nearest neighbors considered according to our experiments. The reported AA thus follows Equation (5) (Yale et al. 2020) and is computed on a subset of true training samples due to time constraints.

**Correlation score.** While the above indicators globally assess the difference between true and generated data distributions, Viñas et al. (2022) propose to compare the moments of both distributions. More precisely, the so-called Pearson correlation score is computed from the correlation matrices *M*_t_ and *M*_g_ associated with true and generated data, as follows:


(6) $$\rho (M_{t},M_{g})=\sum_{i=1}^{d}\sum j=i+1^{d}\frac{\left(M_{i,j;t}-M_{i,j;t}\right)}{\sqrt{\sum_{k=1}^{d}M_{i,k;t}^{2}.\sum_{k=1}^{d}M_{i,k;g}^{2}}}.$$

### 4.2 Goals of experiment

A first question is whether and to which extent a GAN architecture can produce augmented data in transcriptomics with significant impact w.r.t. the above performance indicators.

A second question concerns the impact of using a more stable and expensive data augmentation mechanism, typically based on a WGAN loss [Equation (2)] as opposed to a GAN [Equation (1)].

A third question regards the merits of using an attention-based GAN (Section 3.1.3). Due to memory constraints, the attention architecture needs be restricted to only consider a small subset of the gene pairs. The selection of these pairs can be guided by domain knowledge, e.g. related with PPI, or be randomized.

These questions lead to assess and compare five data augmentation processes, respectively, based on: (i) the random addition of Gaussian noise (with variance 0.1 on normalized data); (ii) mainstream GAN (Goodfellow et al. 2014); (iii) WGAN-GP (Section 3.1.2); (iv) a knowledge-based attention-GAN noted AttGan (Section 3.1.3), where the attention structure selects the pairs of genes with highest dependencies w.r.t. PPI, or gene expression, or both; (v) RandAttGan is defined as a lesion study to investigate whether the potential advantage of AttGAN is due to the knowledge encapsulated in the attention architecture, or to its architecture sparsity. The attention structure in RandAttGAN is defined by randomly permuting the genes and keeping same dependencies and attention mask as in AttGAN. Note that the AttGAN models come in 4 modes depending on whether the attention mask is derived: (i) from gene co-expressions (Co-Exp); (ii) from protein–protein interactions (PPI); (iii) from both (Both); (iv) from a randomized mask (RandAttGAN).

### 4.3 Hyper-parameter settings

For GAN models, the hyper-parameter setting is selected after a grid search, retaining the best one in terms of reverse validation (Section 4.1.1) and unsupervised indicators (Section 4.1.2). For the WGAN-GP, the hyper-parameter setting is selected in the same way (details regarding the methodology and the retained hyper-parameters can be found in Supplementary Appendix SB). For attention-based GANs, additional hyper-parameters include: (i) the attention weight *γ*, fixed, or learned (Zhang et al. 2019); (ii) whether the attention operates from scratch or on a neural net that is pre-trained with no attention. The training time (800 epochs with Adam optimizer) is 45 min for the GAN (respectively, 1 h for the WGAN-GP and 1 h 40 min for the longest of AttGANs) on a NVIDIA A40 GPU with 48 GB of RAM.

### 4.4 Measuring supervised performance indicators

The performance indicators based on the predictive accuracy are computed by training an MLP baseline model, optimized through a Bayesian optimization (Akiba *et al.* 2019). Two problems are considered: a binary (cancer yes/no) and a multiclass one (24 tissue types) classification problem. The hyper-parameter setting for the binary (respectively, multiclassification) MLP includes a hidden layer of size 256 (respectively, 64), a learning rate of 10^−3^ (respectively, 5 × 10^−4^), a batch size of 32, Adam optimizer, ReLU activations, and 40 epochs.

The reverse validation measures are based on training an MLP for 35 epochs with fixed architecture (one hidden layer of size 512), with batch size 32, a learning rate 10^−4^, and Adam optimizer.

The data augmentation schedule proceeds in two ways. Firstly, we consider the accuracy obtained when training the MLP from a fixed number (8000) of generated samples and an increasing number of true samples. Secondly, we consider the accuracy obtained when training the MLP from a fixed number (50 and 100) of true samples and an increasing number of generated samples.

The overall dataset is divided as detailed below. All the classification results presented in this article are computed on a separate test set, never used for the training of the generative models. Mean and standard deviations are computed over five runs with same setting.

### 4.5 Benchmark RNA-seq data

The presented experiments consider the Pan Cancer Genome Atlas (TCGA) (Weinstein et al. 2013). The data underlying this article can be retrieved using the RTCGA package in R and processed following the provided Python code.

After preprocessing, the dataset includes 9749 samples of 20 531 gene expression levels each (release date 28 January 2016). The whole dataset is split into training, validation and testing sets, respectively, including 6499, 1625, and 1625 samples.

The Conditionnal GAN (Section 3.1.1) is structured along four covariates [age, gender, cancer labels (yes/no), and tissue types]. Note that only the observed covariate patterns are considered to condition the generated samples, leaving the investigation of interpolated observed patterns for further work.

## 5 Results

This section reports on the results obtained with the different GAN frameworks, considering the whole set of performance indicators (Table 1).

**Table 1.** Performance indicators for all considered generative models.^a^

Model	Correlation ↑	Precision ↑	Recall ↑	F1 score ↑	FD binary ↓	FD tissue ↓	AA
**GAN**	0.1440	0.803 ± 0.0027	0.0 ± 0.0	0.0 ± 0.0	1506121.82 ± 2617.05	96611.18 ± 98.5	0.9925 ± 0.0022
**WGAN-GP**	**0.9098**	**0.9921 ± 0.0009**	0.4932 ± 0.0024	0.6589 ± 0.0013	**16452.74 ± 1531.79**	638.16 ± 36.17	**0.5893 ± 0.0023**
**RandAttGAN PPI + pretrain**	0.8888	0.912 ± 0.002	0.6481 ± 0.0032	0.7577 ± 0.0025	51254.11 ± 3086.38	1136.43 ± 15.71	0.699 ± 0.0056
**AttGAN PPI + pretrain**	0.8854	0.9088 ± 0.0027	0.6562 ± 0.0046	0.7621 ± 0.0034	94004.31 ± 4115.62	1561.11 ± 46.7	0.683 ± 0.0028
**AttGAN PPI + CoExp + pretrain**	0.8815	0.896 ± 0.0019	0.6437 ± 0.0018	0.7492 ± 0.0018	107155.74 ± 2537.0	2203.53 ± 75.3	0.7021 ± 0.0095
**RandAttGAN PPI + CoExp + pretrain**	0.8904	0.9096 ± 0.0015	0.666 ± 0.0012	**0.769 ± 0.0013**	38941.89 ± 1560.36	1207.58 ± 62.27	0.7057 ± 0.0083
**AttGAN PPI + CoExp + gamma fixed**	0.8621	0.7945 ± 0.0029	**0.7203 ± 0.0028**	0.7556 ± 0.0028	32507.75 ± 1119.21	**556.73 ± 14.2**	0.751 ± 0.0037

a Best results in bold.

### 5.1 GAN models

The GAN models show a main limitation, witnessed by a null recall (Table 1), and blamed on the mode collapse (many true data modes seem to have disappeared from the generated data). The high AA (0.9925 ± 0.0022) suggests that the true and generated data are seamlessly distinguishable. Lastly, Frechet distances measured from binary and tissue MLPs are among the worst (highest) ones out of all the models.

**Figure 1.**
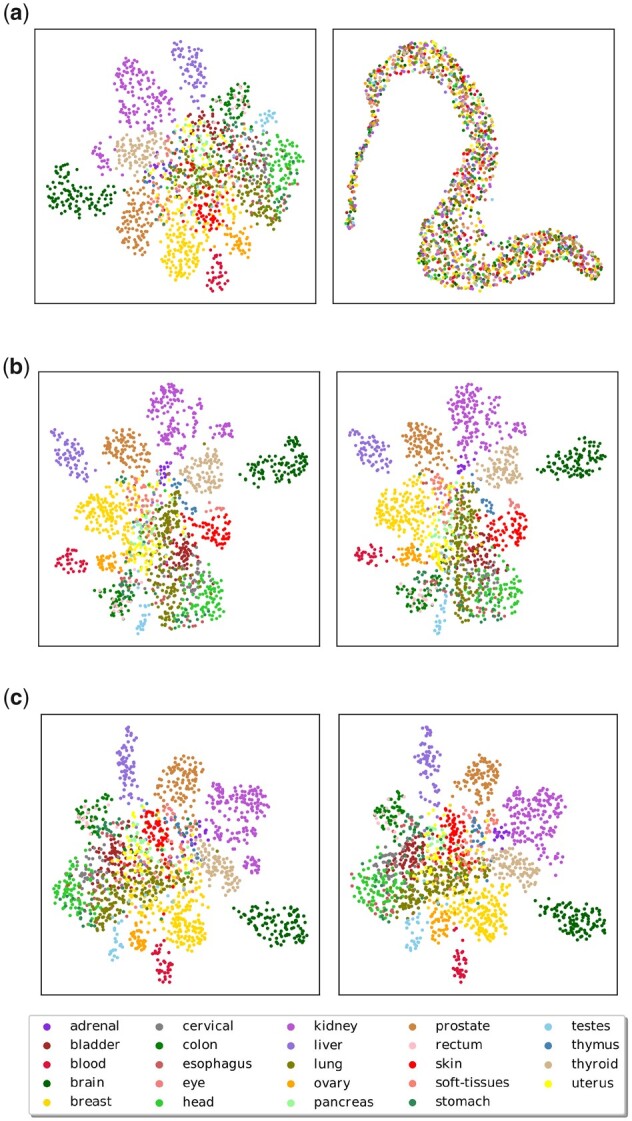
UMAP representation of true validation data (left) and data generated by: (a) our best GAN model; (b) our best WGAN-GP model; (c) our best AttGAN model. Colors highlight different tissue types.

The proposed interpretation in terms of mode collapse is supported by the 2D visualization of the generated data (Fig. 1a), suggesting that the generated data is of lower intrinsic dimension (hardly preserving the tissue patterns) than the true data. The PCA analysis (Fig. 4) confirms this interpretation, showing that the GAN-based generated data are low-dimensional (the top 50 eigenvalues capture the whole variance) compared to the heavy-tailed true data (the top 2000 eigenvalues only capture 90% of the variance).

**Figure 2.**
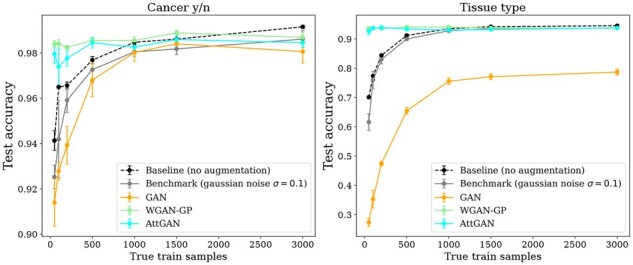
Test accuracy of an MLP trained on an increasing number of true training samples and 8000 augmented samples for both binary (left) and tissue classification (right).

**Figure 3.**
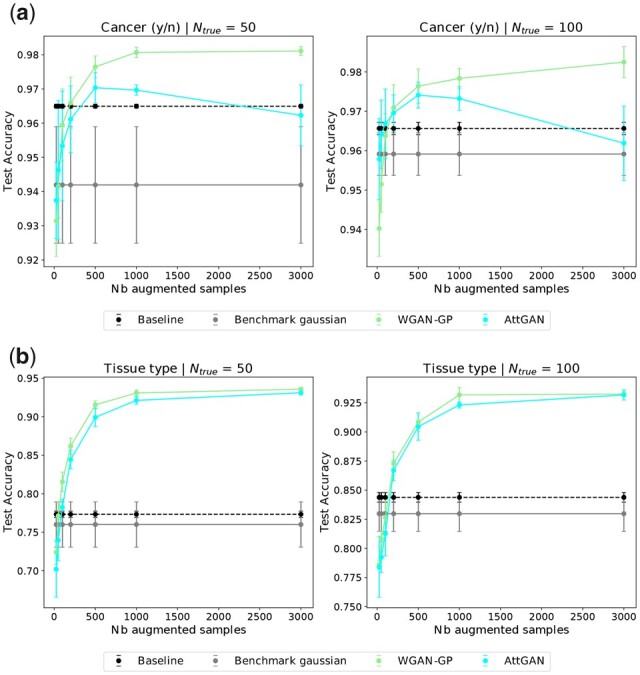
Test accuracy of an MLP trained on 50 (left) and 100 (right) true training samples, with a varying amount *m* of additional augmented data for: (a) cancer yes/no classification; (b) tissue type classification.

The reverse validation (Supplementary Appendix SD) and data augmentation performances (Figs 2 and 3a and b) also show that the GAN generated data provide a lesser discriminant information. In both binary and multiclass prediction cases, augmenting the true data with data generated from the GAN results in a significant accuracy loss (respectively, .03 and .43). The considerable drop in tissue prediction accuracy suggests that the augmented data distribution misses the features supporting the complex interactions involved in this classification task. Eventually, the overall performances are worse than for the Gaussian noise-based augmentation baseline.

### 5.2 WGAN-GP models

For a good hyper-parameter setting, WGAN-GP models generate faithful data (precision 0.9921 ± 0.0009, correlation score .9098, in Table 1), with the best (lowest) FD-binary, and an AA close to 0.5 (0.5893 ± 0.0023).

The limitation of the approach is still witnessed by a low recall (0.4932 ± 0.0024), suggesting that the mode collapse still takes place, yielding a poor coverage of the overall true support. After Fig. 1b, this lack of representativity is not noticeable to the human eye, as true and generated data seem to share very similar dispersion and clusters.

**Figure 4.**
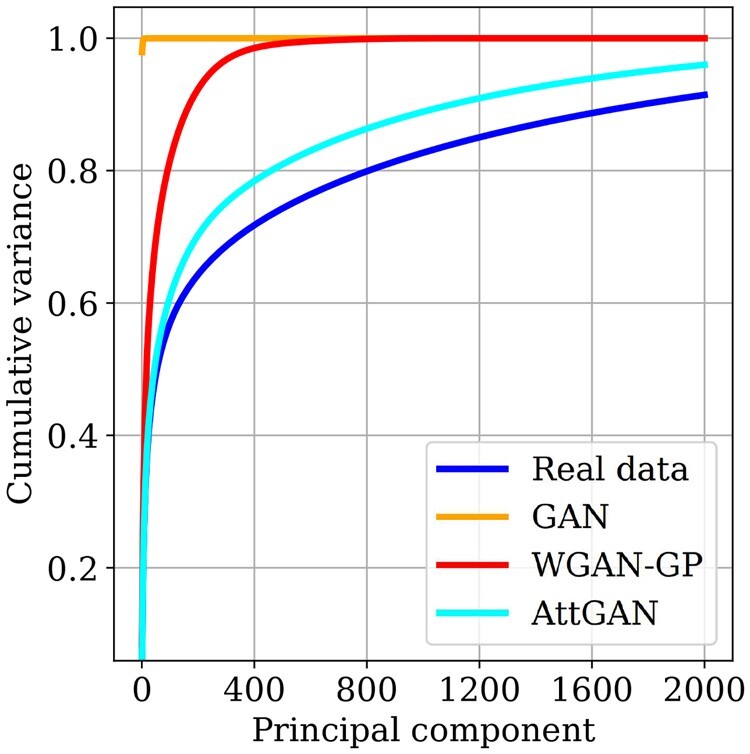
PCA-based comparison of the true, GAN, WGAN-GP, and AttGAN distributions: cumulative variance CV(*i*) explained from the top-*i* principal components.

On one hand, the PCA analysis (Fig. 4) shows that the WGAN-GP generated data live in a much lower dimensional space than the true data (the top-500 eigenvalues capture circa 100% of the variance).

On the other hand, the reverse validation (Supplementary Appendix SD) and data augmentation evaluation (Fig. 2) show that the WGAN-GP does capture some discriminant information, as it is the best model-based augmentation method overall. More precisely, the predictive accuracy jumps from 0.94 to 0.985 in the binary case (from 0.7 to 0.94 in the tissue classification), when adding 3000 generated samples to the 50 true sample training set (Fig. 3a and b).

This advantage decreases and might even become negative when considering a larger number *n* of training samples. For *n *<* *1000, the data augmentation improves the model stability (no regularization is needed). For *n *>* *1000, the accuracy plateaus and adding 8000 generated samples does not improve the performance (Fig. 2). A tentative interpretation is that the generated data fail to capture the fine-grained details of the true data distribution, and might even prevent their detection and exploitation from the true data (making them appearing like noise).

We investigated the optimal number of generated samples depending on the number of true samples. In Fig. 3a, a very fast jump in accuracy is observed when adding a few generated samples. The accuracy thereafter plateaus or very slowly increases after 1000 augmented samples.

### 5.3 AttGAN models

**Figure 5.**
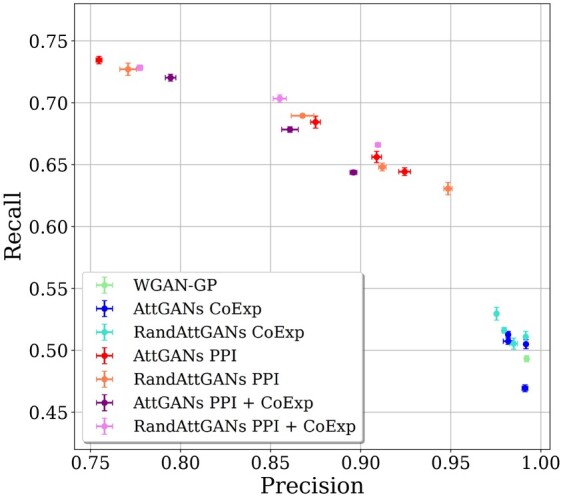
The recall versus precision tradeoff: Pareto frontier obtained for AttGAN with different attention masks (PPI, CoExp, CoExp-PPI, Rand) and different training settings.

The AttGAN models yield a different tradeoff between precision and recall than WGAN-GP, with an average loss in precision of circa 10% and an average gain in recall of circa 15% in Table 1. The general precision-recall tradeoff of all models is displayed in Fig. 5. The configuration with best recall and FD-tissue out of all models is the AttGAN combining both co-expression and PPI knowledge attention heads with a fixed gamma; it has a loss of circa 20% in precision and a gain of circa 23% in recall compared to WGAN-GP.

This AttGAN (CoExp + PPI + gamma fixed) is the one presented in Figs 1c, 2, and 3a and b. It yields significant accuracy improvements (a gain in accuracy of 0.04 for the binary task and 0.24 for tissue prediction) and behaves on par with WGAN-GP (Fig. 2). Surprisingly, the AttGAN-based generated samples cause the binary accuracy to drop (Fig. 3a), when their number increase (*m *>* *500 augmented samples for *n *=* *50 true samples). For tissue type classification, the results are very similar to that of WGAN-GP, and the diversity between generated tissues seems preserved, as illustrated in Fig. 1c.

The overall results suggest that attention mechanisms help data diversity, even if the structure of the attention mask does not significantly matter, be it based on domain knowledge, or on a random mask. The lesion study conducted with RandAttGAN actually shows no impact of the attention mask (Table 1 and Fig. 5). The only difference between RandAttGAN PPI and RandAttGAN CoExp is the size of the random graph (see Supplementary Appendix SA for details on genes interactions/correlations pruning). We thus believe that increasing the co-expression graph size would yield similar results to the ones with PPI knowledge.

Regarding the PCA analysis, Fig. 4 highlights that AttGAN preserves more information with respect to principal components, with a cumulative explained variance at 2000 of 95% (versus 100% for WGAN-GP and 90% for the true data).

## 6 Discussion

A first remark is that the visual analysis, using a UMAP projection, can make differences between poor and good generative models but fails to detect differences between good and (slightly) better models. More precisely, the GAN fails to preserve the clusters associated with tissue types (Fig. 1a), while the clustering structure is preserved by both WGAN-GP and AttGAN (Fig. 1b and c).

A second remark is that the PCA analysis provides a more precise assessment. Figure 4 shows that the GAN-generated distribution is not at all representative of the distribution of the true data; WGAN-GP is better, and AttGAN is much better. Further work will be conducted to compare the true and generative spaces considering the eigenvectors besides the eigenvalues.

A third remark is that the more faithful AttGAN distribution compared to that of WGAN-GP does not necessarily translate into better supervised performance. Likewise, as noted by Devries and Taylor (2017) and Yun et al. (2019) in the context of computer vision, data augmentation with unrealistic samples (images with zero pixels patches or patches combined from different images) can still lead to significant performance improvements. The optimal generative model in terms of performance improvement is domain-dependent. Typically, the data augmentation based on perturbations with Gaussian noise impairs the classification performance in the presented results.

Fourthly, the complementarity and correlations of the indicators are illustrated in Table 1. The best approaches in terms of supervised indicators are WGAN-GP and AttGAN (Fig. 2). These approaches correspond to different tradeoffs between precision and recall (also related to F1 score and AA), with a loss in precision (respectively, a gain in recall) of 10% (respectively, 20%) of AttGAN compared to WGAN-GP. The F1 and FD scores are also correlated with performance improvement. F1 yields an overall similarity indicator w.r.t. the true data in the original feature space. FD measures the similarity w.r.t. a latent space designed for classification; it thus indicates whether the discriminant information has been preserved.

As regards attention, the results of the AttGAN models are promising. They reach comparable scores to that of WGAN-GP at their best, with better PCA, F1, and FD-tissue scores. However, the lesion study (RandAttGAN) shows that the good behavior of AttGAN is not due to using domain knowledge to specify how genes interact. The results of AttGAN can thus only be explained from its more powerful architecture space, enabling the consideration of joint effects of some (random) pairs of genes with flexibility. Further work will consider attention frameworks with limited complexity (Wang et al. 2020; Hawthorne et al. 2022).

Lastly, the relevance of the augmented data w.r.t. cancer/tissue label knowledge is shown (Supplementary Appendix SE; more detailed accuracy results per tissue for our best WGAN-GP and AttGAN models are also displayed in Supplementary Appendix SF). The accuracy is improved for all 24 tissues (except eye and rectum tissues) by augmenting the data with 1000–3000 generated samples. Further work will investigate specific data augmentation strategies, depending on their representativity in the dataset (Supplementary Appendix SG).

## 7 Conclusion

This article comprehensively reviews and evaluates GAN-based generative models on the TCGA dataset in view of achieving data augmentation and enabling deep learning applications to transcriptomics.

The considered approaches include various training criteria and neural architectures of increasing complexities, ranging from GAN to WGAN-GP and AttGAN. In the latter case, several attention structures (based on domain knowledge or randomly drawn with the same representation power) are considered. Each approach is assessed according to the state-of-art performance indicators and new transcriptomics-oriented ones.

The first lesson learned is that performance indicators capture different facets of the generated data that might or not be related to the data augmentation goals. The supervised accuracy indicators depend on precision and recall. Two good configurations are identified. A second lesson is that the benefits of data augmentation depend on the amount of true samples in the training set and tend to plateau or even decrease when too many generated samples are considered (Fig. 2). The proposed interpretation is that by increasing the data density in the well-modeled data modes, massive data augmentation might cause the smallest (true) data modes appear like noise.

Investigating in more depth the relationship between the indicators and the supervised accuracy of data augmentation opens several research perspectives.

Finally, building upon the AttGAN results, an interesting multidisciplinary perspective consists in opening the black box of the random attention module and interpreting what it captures in terms of gene patterns and interactions.

## Supplementary Material

btad239_Supplementary_Data
