# Supplementary material for: GAN-based data augmentation for transcriptomics: survey and comparative assessment
Source: Bioinformatics. 2023 Jun 30;39(Suppl 1):i111–20. doi: 10.1093/bioinformatics/btad239 (PMC10311334; doi:10.1093/bioinformatics/btad239)
Supplement: btad239_Supplementary_Data [file btad239_supplementary_data.pdf]

Appendix A. Attention module

The attention framework was built based on two domain knowledge: protein-protein interactions (PPI) and/or co-expression, namely correlations between genes.

Attention knowledge masks

The level of PPI was extracted from the STRING database[1] where the full protein network data with scored links between proteins is available. Relations between humans genes and proteins are then retrieved from BioMart server[2]. Details of the query to retrieve the TCGA corresponding genes and map the protein-protein interactions to gene-gene interactions are available in the code provided in the paper.

The pairwise Pearson correlations are directly computed on the TCGA training set to retrieve the co-expression information. Both PPI and correlations are then thresholded given a removal percentage (e.g a threshold at 0.9 means 90% of the lowest interactions/correlations are removed). For genes falling into both categories, we compute attention scores with both knowledge heads (PPI and CoExpression) and then compute the final score as a linear combination of both. The parameters (β) of this transformation are initialized as a random convex combination and then learned for each gene (and attention head). We obtain the following weighted sum:

$$x_i = \beta_{i,ppi} att_{ppi}(x_i^{L-1}) + \beta_{i,coexp} att_{coexp}(x_i^{L-1})$$

The choice of the pairs with known relations (either based on protein-protein interactions or pairwise correlations) is a first attempt to select useful pairs of genes. The lesion experiment (section 4.2) however shows that the structure of attention helps, but a random attention mask behaves on par with (or better than) a mask based on domain knowledge.

Threshold	Protein-Protein Interactions (PPI)	CoExpression (CoExp)	PPI genes of interest	CoExp genes of interest
0.7	401 370	132 588	14 044	5 872
0.8	287 970	33 006	12 057	2 648
0.9	199 621	8 014	10 187	**758**
0.95	92 549	3 302	8 369	335
0.98	54 432	1 170	6 591	148
0.99	39 472	526	5 502	99
0.995	29 928	218	**4 670**	55

Table 1. Number of interactions and genes remaining after thresholding. Given thresholds correspond to the amount of lowest interactions/correlations removed from the knowledge graph. As a trade-off between architecture expression and computational time and resources, the number of genes retained for PPI and CoExpression attention masks are highlighted in bold (thresholds of 99.5% and 90% respectively for PPI and CoExpression).

Attention architecture

The attention module is made of three linear layers: a key, query and value layer. Given a subset of genes of interest (selected as the X% of genes having the highest protein-protein interactions or pairwise correlations), the keys, queries and values are computed for all these genes. Then, attention scores based on a similarity function (between keys and queries) are computed pairwise only for the genes interacting with each other in the knowledge graph. The usual computation of e_{ij} (section 3.1.3 in paper) with a cosine is replaced with the absolute difference of (scalar) k_i and q_j to compute the similarity. Pairwise attention scores slow down the computation process but it allows us to manage the computational resources and focus on useful interactions for the data generation. The attention mask determines the pairs of genes (G_i, G_j) such that the value of G_j is "viewed" though the agreement between the key of G_i and the query of G_j. A residual connection between the input and output of the self-attention layer (weighted by the γ parameter) is added to improve the stability of the training.

Appendix B. Grid search methodology and best hyper-parameters

GANs training is sensitive to their hyper-parameters therefore, a grid search was conducted on the hyper-parameters space to define the best configuration for each generative model. The retained hyper-parameters setting was such that: i) it leads the generative model to converge; ii) the unsupervised performance indicators (e.g FD, precision, recall) on the train (not test) sets and reverse validation results on the validation set are satisfactory when compared to results on true TCGA data only. In our experiments, convergence was

[1] STRING database of scored links between proteins.

[2] BioMart Server by the Ensembl project.

reached around 800 epochs for the GAN and WGAN-GP as only marginal evolution of the loss would occur at this point in training. One should note that convergence was more easily reached with the WGAN-GP while the simple GAN was more prone to mode collapse. These findings, alongside time constraints, led us to end the training after 800 epochs. Final models were retrieved at the end and all the performance indicators were computed using these final weights. However, there were no single hyper-parameters setting optimizing all indicators. We thus retained all top-5 settings for each indicator and selected the most frequent one. Here is the list of the hyper-parameters that were evaluated: the optimizer, the batch size, the learning rates of the generator and the discriminator respectively, the depth of the generator and discriminator networks respectively, the number of nodes in each hidden layer, the activation functions between hidden layers (either relu or leaky-relu), batch normalization layers and spectral regularization.

Best architecture for the GAN involves: a batch size of 64, leaky relu activations with slope of -0.1, batch norm layers, discriminator learning rate 10^{-4} and generator learning rate 10^{-3}. The architecture is a three-hidden-layer neural net (256- 512- 1024 nodes) for the Generator and two hidden layers (512-256 nodes) for the Discriminator, with dimension of the Generator input space 128, and an embedding dimension of 2 for tissue conditional variables.

Best architecture for the WGAN-GP involves: a batch size of 256, leaky relu activations with slope of -0.5, same learning rates and same neural architectures as for the best GAN, a lambda parameter at 10, 5 iterations for the Discriminator (for 1 iteration of the Generator). Same neural architecture as for the GAN but batch-norm layers are not retained.

For the **AttGANs**, we retained the best hyper-parameters for the WGAN-GP and **only adjusted its own hyper-parameters (attention γ parameter, presence of pre-training without attention, type of attention mask)**. The top-5 hyper-parameters combinations (selected as the most frequent ones in the top-5 configurations for each performance indicator) are reported in Table 1 in the paper.

Appendix C. Precision and Recall with k nearest neighbors: how to choose k

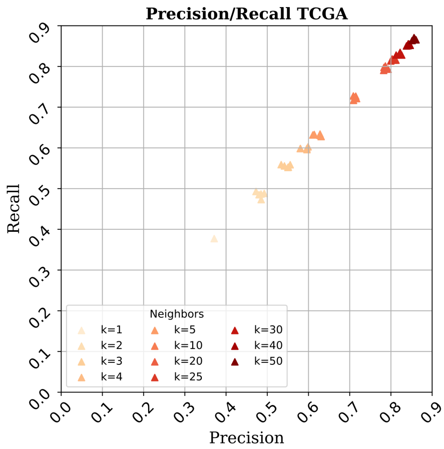

Fig. 1: Precision and recall values computed (5 times) between random batches of 4,000 true samples, depending on k number of nearest neighbors.

Figure 1 shows the evolution of precision and recall indicators on two batches of true TCGA data depending on k number of nearest neighbors used to approximate the true and generated manifolds. Considering we would expect high values (close to 1.) of precision and recall between true samples, we observe such values are obtained only for higher k number of neighbors. Empirically, we find that $k =$ 50 returns satisfying precision and recall values on true data (0.85 for both precision and recall). Thus, this would serve as benchmark precision/recall values to compare to the ones obtained when considering generated data. As shown by Kynkaanniemi et al. (2019), their precision and recall metrics are comparable between models as long as the same k is used. Decreasing or increasing the number of neighbors only performs a translation of overall metrics values.

Appendix D. Reverse validation

Model	Test accuracy cancer (y/n)	Test accuracy tissue type
**GAN**	0.8228 ±0.007	0.0856 ±0.0028
**WGAN-GP**	0.9839 ±0.0022	0.9361 ±0.0027
**RandAttGAN PPI + pretrain**	0.9858 ±0.0013	0.9333 ±0.0019
**AttGAN PPI + pretrain**	0.9826 ±0.0017	0.934 ±0.0038
**AttGAN PPI + CoExp + pretrain**	0.9811 ±0.0033	0.9276 ±0.0034
**RandAttGAN PPI + CoExp + pretrain**	0.9812 ±0.0039	0.9334 ±0.0025
**AttGAN PPI + CoExp + gamma fixed**	0.9843 ±0.0009	0.9361 ±0.0026
**Baseline on true data**	**0.9916 ± 0.0015**	**0.9469 ± 0.0029**

Table 2. Reverse validation results: test accuracy of binary and multiclass MLPs trained on 8000 generated samples only and tested on true TCGA test set. Although a MLP trained on generated data only does not outperform the state-of-the-art resuts (baseline results on last row), it reaches very close accuracy results with data generated by the WGAN-GP and the five AttGANs versions.

Appendix E. Label knowledge preservation in generated data

Model	Test accuracy cancer (y/n)	Test accuracy tissue type
**GAN**	0.9296	0.092
**WGAN-GP**	0.9823	0.9621
**RandAttGAN PPI + pretrain**	0.9867	0.969
**AttGAN PPI + pretrain**	0.9847	0.969
**AttGAN PPI + CoExp + pretrain**	0.9852	0.9695
**RandAttGAN PPI + CoExp + pretrain**	0.9838	0.9646
**AttGAN PPI + CoExp + gamma fixed**	0.9877	0.9621

Table 3. Test accuracy of binary and multiclass MLPs pretrained on true TCGA data and tested on generated data. We observe that the pretrained MLPs are able to correctly label the data generated by the WGAN-GP and the five AttGANs versions (with a higher test accuracy of 96% than the 94% accuracy on true data for tissue classification). However, the accuracy drops for both binary and tissue classification with data generated by the GAN. It seems the generated data does not violate cancer/tissue information except for the GAN.

Appendix F. Data augmentation results per tissue type

Tissue	$N_{true} = 50$	$N_{true} = 50$ $N_{fake} = 1000$	$N_{true} = 50$ $N_{fake} = 3000$	$N_{true} = 100$	$N_{true} = 100$ $N_{fake} = 1000$	$N_{true} = 100$ $N_{fake} = 3000$
**adrenal**	0.81 +/- 0.1245	0.84 +/- 0.0822	**0.9 +/- 0.0**	0.53 +/- 0.329	0.84 +/- 0.0548	**0.9 +/- 0.0**
**bladder**	0.4838 +/- 0.2805	0.9459 +/- 0.027	**0.9919 +/- 0.0121**	0.6243 +/- 0.3283	0.9378 +/- 0.0296	**0.9838 +/- 0.0176**
**blood**	0.9172 +/- 0.0866	0.9448 +/- 0.0393	**0.9793 +/- 0.0463**	0.8966 +/- 0.0	0.9034 +/- 0.0154	**0.9793 +/- 0.0308**
**brain**	0.9133 +/- 0.1097	0.98 +/- 0.0	**0.9813 +/- 0.003**	0.9627 +/- 0.0265	**0.98 +/- 0.0**	**0.98 +/- 0.0**
**breast**	0.8 +/- 0.0726	0.9853 +/- 0.0072	**0.9948 +/- 0.0019**	0.8268 +/- 0.1376	0.9887 +/- 0.0039	**0.9931 +/- 0.0024**
**cervical**	0.4323 +/- 0.2999	0.8516 +/- 0.0289	**0.8581 +/- 0.0331**	0.5323 +/- 0.375	**0.8452 +/- 0.0334**	0.8387 +/- 0.0411
**colon**	0.5846 +/- 0.2851	0.9754 +/- 0.0084	**0.9846 +/- 0.0**	0.7108 +/- 0.2659	**0.9785 +/- 0.0084**	**0.9785 +/- 0.0084**
**esophagus**	0.2103 +/- 0.2289	**0.4308 +/- 0.4203**	0.1436 +/- 0.2165	0.1641 +/- 0.3004	**0.3897 +/- 0.2611**	0.1282 +/- 0.2019
**eye**	**0.8824 +/- 0.2038**	0.6 +/- 0.5477	0.0 +/- 0.0	**1.0 +/- 0.0**	0.2 +/- 0.4472	0.4 +/- 0.5477
**head**	0.7453 +/- 0.2219	**0.9245 +/- 0.0353**	0.9189 +/- 0.042	0.8547 +/- 0.0659	0.9302 +/- 0.0171	**0.9415 +/- 0.0155**
**kidney**	0.7586 +/- 0.201	**0.9859 +/- 0.002**	0.9841 +/- 0.0024	0.8925 +/- 0.1138	**0.9877 +/- 0.0057**	0.9815 +/- 0.0072
**liver**	0.8378 +/- 0.0469	0.9422 +/- 0.0093	**0.9533 +/- 0.005**	0.8511 +/- 0.0617	0.9467 +/- 0.0145	**0.9489 +/- 0.0061**
**lung**	0.4141 +/- 0.2504	**0.9621 +/- 0.0106**	0.9568 +/- 0.0079	0.726 +/- 0.0917	**0.9604 +/- 0.0128**	0.948 +/- 0.0141
**ovary**	0.6281 +/- 0.2778	**1.0 +/- 0.0**	**1.0 +/- 0.0**	0.9754 +/- 0.0457	0.993 +/- 0.0157	**0.9965 +/- 0.0078**
**pancreas**	0.551 +/- 0.2126	0.9551 +/- 0.0266	**0.9592 +/- 0.0144**	0.6327 +/- 0.3201	0.9306 +/- 0.0423	**0.9388 +/- 0.0204**
**prostate**	0.8695 +/- 0.1879	**1.0 +/- 0.0**	0.9983 +/- 0.0038	0.9898 +/- 0.0071	**1.0 +/- 0.0**	0.9983 +/- 0.0038
**rectum**	**0.2273 +/- 0.085**	0.0 +/- 0.0	0.0 +/- 0.0	**0.2 +/- 0.3349**	0.0 +/- 0.0	0.0 +/- 0.0
**skin**	0.628 +/- 0.2728	0.9634 +/- 0.0163	**0.9677 +/- 0.0**	0.8323 +/- 0.1296	**0.9742 +/- 0.0096**	0.9677 +/- 0.017
**soft-tissues**	0.6226 +/- 0.183	0.9226 +/- 0.0528	**0.9323 +/- 0.0265**	0.4 +/- 0.2463	**0.9484 +/- 0.0135**	0.929 +/- 0.0144
**stomach**	0.4088 +/- 0.3586	0.5912 +/- 0.3619	**0.8618 +/- 0.1707**	0.5265 +/- 0.2841	0.5529 +/- 0.2331	**0.8529 +/- 0.1995**
**testes**	0.8529 +/- 0.11	0.8941 +/- 0.0395	**0.9235 +/- 0.0161**	0.8294 +/- 0.0868	0.9 +/- 0.0492	**0.9 +/- 0.0161**
**thymus**	0.725 +/- 0.282	0.85 +/- 0.0559	**0.8583 +/- 0.0559**	0.8083 +/- 0.1126	0.825 +/- 0.0456	**0.8333 +/- 0.051**
**thyroid**	0.923 +/- 0.0598	**0.9918 +/- 0.0058**	0.9852 +/- 0.0107	0.9705 +/- 0.017	**0.9967 +/- 0.0045**	0.9885 +/- 0.0093
**uterus**	0.6739 +/- 0.1969	0.9435 +/- 0.0364	**0.9435 +/- 0.033**	0.6652 +/- 0.2083	0.9174 +/- 0.0677	**0.9522 +/- 0.0283**

Table 4. Test accuracy per tissue type after training a MLP (tissue type classification) on either 50 or 100 true samples (N_{true}) and 0, 1000 and 3000 augmented samples (N_{fake}) generated by our best WGAN-GP model. Best accuracy (given a number of true samples N_{true}) in bold.

**Tissue**	$N_{true} = 50$	$N_{true} = 50$ $N_{fake} = 1000$	$N_{true} = 50$ $N_{fake} = 3000$	$N_{true} = 100$	$N_{true} = 100$ $N_{fake} = 1000$	$N_{true} = 100$ $N_{fake} = 3000$
**adrenal**	0.81 ± 0.1245	**0.9 ± 0.0**	0.84 ± 0.0652	0.53 ± 0.329	0.88 ± 0.0447	**0.89 ± 0.0224**
**bladder**	0.4838 ± 0.2805	0.9324 ± 0.0214	**0.9568 ± 0.0113**	0.6243 ± 0.3283	0.9378 ± 0.0154	**0.9486 ± 0.041**
**blood**	0.9172 ± 0.0866	0.9517 ± 0.0523	**0.9724 ± 0.0289**	0.8966 ± 0.0	0.9586 ± 0.045	**0.9862 ± 0.0308**
**brain**	0.9133 ± 0.1097	**0.98 ± 0.0**	0.9787 ± 0.003	0.9627 ± 0.0265	**0.9787 ± 0.003**	**0.9787 ± 0.003**
**breast**	0.8 ± 0.0726	**0.9913 ± 0.0043**	0.9905 ± 0.0036	0.8268 ± 0.1376	0.9896 ± 0.0084	**0.9922 ± 0.0056**
**cervical**	0.4323 ± 0.2999	**0.8677 ± 0.021**	0.8516 ± 0.0177	0.5323 ± 0.375	0.8516 ± 0.031	**0.8935 ± 0.0088**
**colon**	0.5846 ± 0.2851	0.92 ± 0.1204	**0.9815 ± 0.0069**	0.7108 ± 0.2659	**0.9846 ± 0.0**	0.9785 ± 0.0084
**esophagus**	0.2103 ± 0.2289	**0.4103 ± 0.1612**	0.1795 ± 0.1612	0.1641 ± 0.3004	**0.5231 ± 0.3375**	0.2821 ± 0.2257
**eye**	**0.8824 ± 0.2038**	0.6 ± 0.5477	0.5882 ± 0.5375	**1.0 ± 0.0**	0.6 ± 0.5477	0.3529 ± 0.4904
**head**	0.7453 ± 0.2219	0.9302 ± 0.0338	**0.9453 ± 0.0301**	0.8547 ± 0.0659	0.8925 ± 0.028	**0.9509 ± 0.0205**
**kidney**	0.7586 ± 0.201	0.9841 ± 0.0024	**0.9877 ± 0.0048**	0.8925 ± 0.1138	**0.9877 ± 0.002**	0.9859 ± 0.002
**liver**	0.8378 ± 0.0469	0.94 ± 0.0186	**0.9556 ± 0.0**	0.8511 ± 0.0617	**0.9533 ± 0.005**	0.9533 ± 0.0122
**lung**	0.4141 ± 0.2504	**0.9507 ± 0.0079**	0.9498 ± 0.0158	0.726 ± 0.0917	**0.963 ± 0.0101**	0.9436 ± 0.0223
**ovary**	0.6281 ± 0.2778	**1.0 ± 0.0**	0.993 ± 0.0096	0.9754 ± 0.0457	**1.0 ± 0.0**	0.9895 ± 0.0157
**pancreas**	0.551 ± 0.2126	0.9306 ± 0.0341	**0.9714 ± 0.0112**	0.6327 ± 0.3201	**0.9388 ± 0.0456**	0.9184 ± 0.0433
**prostate**	0.8695 ± 0.1879	**1.0 ± 0.0**	0.9966 ± 0.0076	0.9898 ± 0.0071	**1.0 ± 0.0**	0.9949 ± 0.0076
**rectum**	**0.2273 ± 0.085**	0.1364 ± 0.2802	0.0 ± 0.0	**0.2 ± 0.3349**	0.0 ± 0.0	0.0 ± 0.0
**skin**	0.628 ± 0.2728	0.9699 ± 0.0118	**0.9785 ± 0.0108**	0.8323 ± 0.1296	0.9656 ± 0.0177	**0.9785 ± 0.0076**
**soft-tissues**	0.6226 ± 0.183	**0.9581 ± 0.0216**	0.9323 ± 0.0385	0.4 ± 0.2463	0.9387 ± 0.0502	**0.9581 ± 0.0184**
**stomach**	0.4088 ± 0.3586	0.6176 ± 0.1348	**0.8353 ± 0.1197**	0.5265 ± 0.2841	0.4676 ± 0.3426	**0.7382 ± 0.1694**
**testes**	0.8529 ± 0.11	**0.9235 ± 0.0161**	0.9118 ± 0.036	0.8294 ± 0.0868	0.9118 ± 0.0509	**0.9118 ± 0.0208**
**thymus**	0.725 ± 0.282	0.8167 ± 0.0475	**0.8667 ± 0.0349**	0.8083 ± 0.1126	0.825 ± 0.0349	**0.8333 ± 0.051**
**thyroid**	0.923 ± 0.0598	**0.9967 ± 0.0045**	0.9885 ± 0.011	0.9705 ± 0.017	**0.9951 ± 0.0045**	0.9918 ± 0.0058
**uterus**	0.6739 ± 0.1969	**0.9261 ± 0.0607**	0.9043 ± 0.0917	0.6652 ± 0.2083	**0.9435 ± 0.0451**	0.9391 ± 0.0283

Table 5. Test accuracy per tissue type after training a MLP (tissue type classification) on either 50 or 100 true samples (N_{true}) and 0, 1000 and 3000 augmented samples (N_{fake}) generated by our best AttGAN model. Best accuracy (given a number of true samples N_{true}) in bold.

Appendix G. Tissue repartition

Tissue	Proportion in train (%)	Proportion in test (%)
adrenal	0.73	0.98
bladder	4.35	3.64
blood	1.96	1.43
brain	6.70	7.38
breast	12.08	11.37
cervical	3.04	3.05
colon	3.21	3.20
esophagus	1.93	1.92
eye	0.78	0.84
head	5.65	5.22
kidney	12.03	11.17
liver	4.64	4.43
lung	11.82	11.17
ovary	3.08	2.81
pancreas	1.65	2.41
prostate	5.32	5.81
rectum	1.02	1.08
skin	4.58	4.58
soft-tissues	2.49	3.05
stomach	2.56	3.35
testes	1.29	1.67
thymus	1.19	1.18
thyroid	5.49	6.00
uterus	2.44	2.26

Table 6. Tissue labels repartition in training set (used for training generative models and baseline MLPs on true data) and true test set used to compute all supervised indicators in the paper.
